# Cognitive strategy in verbal fluency: sex differences, menstrual cycle, and menopause effects

**DOI:** 10.1007/s10339-025-01265-w

**Published:** 2025-04-05

**Authors:** Patricia E. Cowell, Meghana Wadnerkar Kamble, Ramya Maitreyee, Rosemary A. Varley

**Affiliations:** 1 School of Allied Health Professions, Nursing and Midwifery, University of Sheffield, Sheffield, UK; 2 School of Health Sciences, University of East Anglia, Norwich, UK; 3 Institute of Health Sciences, Bhubaneswar, India; 4 Division of Psychology and Language Sciences, University College London, London, UK

**Keywords:** Verbal fluency, Sex differences, Menstrual cycle, Menopause, Cognitive strategy, Language

## Abstract

Cognitive sex differences are shaped by hormone effects on brain development, organisation, structure, function, and ageing. In human speech and language, sex differences and hormone effects are typically studied in the form of performance-based differences (via measures of central tendency) with little attention given to underlying cognitive strategy. This study presents data from 126 healthy adults, aged 20–79 years, from three studies of letter based verbal fluency. Comparisons were conducted based on sex, menstrual cycle phase, and menopause stage to examine total words produced, plus switching and clustering strategy use. The investigation probed differences in performance, underlying cognitive strategies, and correlations between performance and strategy. For performance, there were no statistically significant sex or menopause group differences in total words, number of switches and cluster size. Menstrual cycle differences were significant for switches and cluster size, but not total words. However, there were large effect sizes for correlations between total word performance and strategy measures in some groups; these correlations formed patterns which differed as a function of sex, menstrual cycle phase, and menopausal stage. Words produced were highly correlated with switching in younger women at higher hormone menstrual cycle phases. Correlations between total words and both strategies were moderate and equivalent in older premenopausal and perimenopausal women. Postmenopausal women showed a pattern of higher correlation between total words and cluster size which was observed in younger women at the lower hormone cycle phase, and men. This study illustrates the impact of hormones and sex differences on strategy use in verbal fluency—underscoring the value of comparisons in strategy use between women at different reproductive life stages.

**Supplementary Information:**

The online version contains supplementary material available at 10.1007/s10339-025-01265-w.

## Introduction

The brain is both a target for and a source of sex hormones (Acaz-Fonseca et al. 2016; Lu et al. 2019; McEwen and Milner 2017; Zarate et al. 2017) with diverse impacts on human behaviour and cognition. Hormones exert their effects on neural systems across the lifespan, shaping behaviour and cognition through changes in gene expression (Calvo et al. 2016), neuronal function (Barth et al. 2015; Zarate et al. 2017), neural networks (Ali et al. 2018), and, in later life, neuroinflammation (Sarvari et al. 2012). In early life, hormones are involved in the early differentiation of female and male brains, and in the active feminisation and masculinisation of behaviour and cognition into adolescence. Hormones continue to shape neurocognitive structure and function across the adult years (Jacobs and Goldstein 2018; Rehbein et al. 2021; Ruehr et al. 2024), and have a critical role in sex differences in brain aging (Gurvich et al. 2018). In later life, estrogen, progesterone and testosterone impact neuronal (Zarate et al. 2017) and microglial (Nissen 2017) aging and neural disease progression. Thus, men and women show different patterns of healthy cognitive aging and decline (Gurvich et al. 2018; Levine et al. 2021; Matthews et al. 2016; McCarrey et al. 2016; Nebel et al. 2018; Rahman et al. 2019; Taylor et al. 2019). The neuroprotective effects of estrogen throughout adulthood have also been documented at the systems, synaptic, cellular and molecular levels (Zarate et al. 2017). In turn, these neuroprotective effects have been evaluated in relation to cortical aging and cognitive status in older adults (Kelly et al. 2008; Taylor et al. 2019).

Across the adult life span in women, naturally occurring changes in reproductive phases and stages allow for investigation of the dynamic interplay of hormonal and neurocognitive events. In adult women of reproductive age, the influence of hormones on the female brain, communicative behaviour and related cognitive function has been documented via human (Fernandez et al. 2003; Gurvich et al. 2018; Hamson et al. 2016; Kantarci et al. 2018; Kilpi et al. 2020; Reuben et al. 2021; Ryan et al. 2009; Weber et al. 2014) and animal models (Kelley et al. 2020; Tobiansky and Fuxjager 2020; Vahaba and Remage-Healey 2018). In terms of sex differences in humans, female advantages in the verbal domain are well documented (Barel and Tzischinsky 2018; Halpern and LaMay 2000; Hyde 2016; Yonker et al. 2003), and present a starting point from which to investigate the impact of ovarian hormones on speech and language abilities. Reproductive cycle models (e.g., menstrual and estrus) have been applied to capture the changes that occur over time in adult females from various mammalian species (Dubol et al. 2021; Souza et al. 2012; Sundström Poromaa and Gingnell 2014; Warren and Juraska 1997; Woolley and McEwen 1993). In humans, comparisons between menstrual cycle days that correspond to high or low ovarian hormone levels have revealed phasic effects on speech and language-based tasks (Scheuringer and Pletzer 2017; Wadnerkar et al. 2006; Whiteside et al. 2004). Some reports investigated changes in performance or ability associated with phase-specific hormone levels (Hampson 1990b, a; Mordecai et al. 2008; Rosenberg and Park 2002); whereas, others documented shifts in functional lateralisation (Cowell et al. 2011; Wadnerkar et al. 2008a; Wong-Goodrich et al. 2020). Meta-analyses have shown significant, yet small, effect sizes for sex differences in verbal episodic memory, verbal fluency (Hirnstein et al. 2022; Hyde 2016) and dichotic listening (Voyer 2011). While not all studies report significant sex differences (Kheloui et al. 2023) or menstrual cycle effects (Pletzer et al. 2024; Sundström Poromaa and Gingnell 2014) in relation to verbal behaviours, evidence indicates that there may be a more consistent degree of female advantage on neurocognitive systems that involve phonetic cueing in speech perception and production (Sato 2020).

Whilst gonadal hormones impact on differences in performance to varying degrees, inter-task correlations also vary between the sexes (Cowell and Hugdahl 2000). This approach to characterising the impact of hormones on behaviour and cognition via the associations among tasks or task components, brings a neuropsychological element to the study of hormone effects. By comparing patterns of correlations across the sexes and, importantly, within the sexes across hormonal states or reproductive life stages (Cowell 2010), one can statistically model and probe differences in functional organisation. Such correlational models can also provide conceptual insight into cognitive networks (i.e., cross-task analysis) and cognitive strategies (i.e., within-task component analysis).

Verbal fluency is a multifactorial (Troyer et al. 1997), efficient and non-invasive test (Melinder et al. 2005) commonly used in cognitive neuropsychological assessments (Patterson 2011). Verbal fluency also has attributes that makes it an ideal measure of interest in the study of cognitive ageing, cognitive strategy use, hormone effects and sex differences. The verbal fluency test provides a brief observation of verbal functioning and is commonly administered as a category/semantic (Benton 1968) or phonemic/letter-based task (Newcombe 1969). Studies using verbal fluency to study sex and hormone effects have typically used the letter-based fluency task, where participants are required to generate words beginning with a particular letter in a given time span. Then, the total number of words generated is used to assess word count, minus any repetitions and errors. An additional scoring technique involves coding for specific retrieval patterns, namely cluster size and number of switches made between clusters (Troyer et al. 1997). Clustering in the letter-based task involves production of strings of words starting with the same letter combinations, whereas switching involves search processes and cognitive set shifting to end clusters and start new ones (Hirshorn and Thompson-Schill 2006; Rende et al. 2002; Temple 2002; Troyer et al. 1997, 1998a, b). The total words produced provides a quantitative indicator of overall performance, whereas looking at the cluster size and number of switches provides indicators of the underlying cognitive strategies employed in the fluency task. In theory, efficient performance on the verbal fluency task involves optimisation of clustering and/or switching.

Verbal fluency has been used to demonstrate the effects of sex differences (Costa et al. 2014; Hirnstein et al. 2022; Hyde 2016; Scheuringer and Pletzer 2017; Weiss et al. 2006), the menstrual cycle (Hampson 1990a), the menopause (Berent-Spillson et al. 2012; Weber et al. 2014), and sex chromosome conditions such as Turner’s Syndrome (Temple 2002). In these studies, the letter-based verbal fluency task was a sensitive indicator of the hormonal impact on cognition in women. However, in keeping with the larger literature on sex and hormone effects on verbal abilities, not all reports on verbal fluency showed sex (Scheuringer and Pletzer 2017; Sokołowski et al. 2020), menstrual phase (Pletzer et al. 2024; Sundström Poromaa and Gingnell 2014), or menopause-associated hormone effects (Herlitz et al. 2007; Kilpi et al. 2020; Kocoska-Maras et al. 2011). In relation to healthy ageing, the relative stability of overall verbal fluency performance for the letter or phonemic version of the task (Troyer et al. 1997), compared to its semantic fluency counterpart (Elgamal et al. 2011; Maitreyee et al. 2023), confers its additional suitability for comparisons across age groups where hormonally based effects are the focal design feature (Berent-Spillson et al. 2012). To probe verbal fluency more deeply, analyses of clustering and switching may provide a more sensitive set of measures for examining the effects of sex, reproductive hormones, and gender specific life stages on the cognitive strategies that underly performance. Strategy-based differences between the sexes (Weiss et al. 2006) and across phases of the menstrual cycle (Scheuringer and Pletzer 2017) have been previously studied by way of verbal fluency clustering and switching and their neurofunctional correlates (Scheuringer et al. 2020). The statistical relationships between total words generated and switching *versus* clustering strategies have been studied in relation to healthy aging (Troyer et al. 1997) and, more recently refined in the context of task- and sex-based effects (Scheuringer et al. 2017). We extend this approach to women across menstrual cycle phases and menopausal stages in the current research.

Verbal fluency is subserved by lateralised frontotemporal systems. Frontal cortex including the left inferior frontal gyrus is implicated in phonological processing, verbal working memory (Costafreda et al. 2006) and the phonological demands required for letter-based fluency (Baldo et al. 2006). The left frontal cortex is also implicated in motor-speech behaviours (Bohland and Guenther 2006; Riecker et al. 2005). While switching is subserved by the left frontal regions, clustering is subserved by the left temporal cortex (Hirshorn and Thompson-Schill 2006; Rende et al. 2002; Troyer et al. 1997, 1998a, 1998b). Moreover, the cortical regions linked to verbal fluency are reported to be sensitive to estrogenic actions (Dietrich et al. 2001; Hamson et al. 2016; Kantarci et al. 2018) and ageing effects (Heinzel et al. 2013; Kahlaoui et al. 2012). The sensitivity of the neurocognitive systems that underpin verbal fluency, to the interactive effects of hormone and age effects, further strengthens the case for conducting a detailed exploration of clustering and switching strategies in relation to hormone phases and reproductive stages in women.

The current paper reports verbal fluency results of three studies from our laboratory, with a focus on the profiles of underlying cognitive strategies in verbal fluency as compared between: (a) men and women (ages 20–27 years), (b) women in different menstrual cycle phases (ages 20–27 years) and (c) menopausal stages (ages 47–79 years). Combining data in this way allowed for greater sample sizes and enabled comparisons that were central to investigating the effects of ovarian hormones on women’s cognitive processing as a function of reproductive life stage. Traditional measures of verbal fluency performance such as total words produced were investigated. In addition, a detailed examination of verbal fluency strategies based on Troyer et al.’s (1997) clustering and switching was conducted. Importantly, the relationships between clustering, switching and total word production were compared across the sexes, and in women across menstrual cycle phases and menopausal stages. The analysis was designed to uncover differences in the way hormones affect verbal behaviour by how they influence its underlying cognitive organisation in healthy adult men and women through a detailed comparison of correlation patterns between the sexes, menstrual cycle phases, and menopausal stages. The study’s primary objective was to investigate verbal fluency performance in relation to the fluid cognitive processing abilities that subserve rapid spoken word retrieval. This study specifically set out to investigate cognitive verbal fluency strategy measures and their correlations with overall performance. These involve clustering strategies in relation to lexical retrieval of word forms with common orthographic/phonemic onsets and switching to different clusters, reflecting a higher order executive function involved in breaking sets (i.e., abandoning a current form-based cluster that has become unproductive, and moving to a new cluster). Thus, we posed the following questions: (i) how will the total word count be affected by sex differences, menstrual phases and menopausal stages; (ii) how will verbal fluency strategies, i.e., clustering and switching, be affected by sex differences, menstrual phases and menopausal stages; and (iii) what are the relationships between clustering, switching and total word production and do these relationships differ as a function of sex, menstrual phase and menopausal stage?

The study set out to test three hypotheses:

i) Total word production would be greater in women compared to men, in women during the high hormonal phase compared to women during the low hormonal phase of the menstrual cycle, and in pre/perimenopausal women compared to post-menopausal women.
ii) Total switches would be greater in women compared to men, in women during the high hormonal phase compared to women during the low hormonal phase of the menstrual cycle, and in pre/perimenopausal women compared to post-menopausal women.

It was also hypothesised that (iii) total word production would be more highly correlated with number of switches in women compared to men, in women during the high hormonal phase compared to women during the low hormonal phase of the menstrual cycle, and in pre/perimenopausal women compared to post-menopausal women. This hypothesis was based on growing evidence for the role of ovarian hormones in women’s executive function (Dunkin et al. 2005; Erickson et al. 2007; Navarro-Pardo et al. 2018; Sundström Poromaa and Gingnell 2014) which the current study sought to investigate via switching as an indicator of flexible cognitive processing in verbal fluency (Hamson et al. 2016).

## Methods

### Participants

The main methods and design features of the included studies are described below and summarised in Table 1.

**Table 1:** Designs used in the current report, plus summary statistics for sex, age, hormone status, handedness and FIQ for the two datasets

Study design and comparisons	Age range mean (SE)	Women’s hormone status expressed as testing day of menstrual cycle or menopause stage	Handedness median (range)	FIQ range mean (SE)
Menstrual cycle and sex differences N = 55	20–27 years, M = 22.58 (0.27)	Days of testing, Mean (SE)	All: + 34 (+ 8 to + 40)	All: 103–138 119.67 (1.13)
Sex: Male vs. Female Cycle Phase: Menstrual vs. Periovulatory/Midluteal HEP Women n = 18 LEP Women n = 17 Men n = 20	HEP Women: 23.22 (0.47) LEP Women: 22.41 (0.56) Men: 22.15 (0.38) F(2, 52) = 1.452, n.s	HEP Women, Day 8–25, 18.94 (1.25) LEP Women*, Day 2–5, 4.06 (0.26)	HEP Women: + 32 (+ 8 to + 38) LEP Women: + 35 (+ 12 to + 40) Men: + 34 (+ 28 to + 39)	HEP Women: 119.83 (2.24) LEP Women: 116.71 (1.88) Men: 122.05 (1.66) F(2, 52) = 1.93, n.s
Menopause N = 71	47–79 years Mean = 60.08 (1.14)	Premenopause: monthly cycles, but with subtle changes in flow and cycle length	All: + 100 (− 100 to + 100)	All: 103–132 120.63 (0.85)
Menopause stage: Pre/Perimenopausal vs Postmenopausal Women Pre- and Perimenopause n = 19	Pre- and Perimenopause Women, 47–55 years: 50.32 (0.44)	Perimenopause: irregular cycles with delays in occurrence	Pre- and Perimenopause Women: + 90 (− 100 to + 100)	Pre- and Perimenopause Women: 118.37 (1.69)
Postmenopause n = 52	Postmenopause women 48–79 years: 63.65 (1.21) F(1,69) = 43.36, *p* < 0.001	Postmenopause: permanent cessation of menstruation for at least 12 consecutive months	Postmenopause Women: + 100 (− 100 to + 100)	Postmenopause Women: 121.46 (0.97) F(1,69) = 2.66, n.s

SE standard error; HEP High estrogen phase; LEP low estrogen phase; *one woman in this group was tested on day 6. See Methods for details of the two scales used to assess handedness

Participants were healthy women (N = 106) and men (N = 20) with letter fluency data who volunteered for studies on sex differences, menstrual cycle and menopause effects on speech, language, and related behaviour. Participants were recruited from the University of Sheffield student and staff population, as well as the local community, by means of posters, and email circulation. Background demographics, reproductive history, WASI Full IQ (FIQ) scores (Wechsler 1999), handedness, and verbal fluency measures were available for all participants.

Participants were native speakers of English with no known history of neurological, speech, language, or hearing difficulties. Women were not using oral contraceptives, HRT, or any hormone-based medications and had not been pregnant or lactating for a minimum of one year prior to the study.

FIQ was used as a measure of general cognitive ability. Handedness was used to confirm that the sample profiles were consistent with the wider population norms. This measure is salient given the relationship between handedness and neurocognitive organisation of language (Pujol et al. 1999) including verbal fluency in women (Cowell and Gurd 2018; Gurd et al. 2013). Assessment of FIQ and handedness enabled cross-study comparisons in relation to these sample characteristics.

Handedness was measured using a behavioural test battery of: (a) twenty items yielding scores between − 40 (completely left-handed) and + 40 (completely right-handed) in the sex difference and menstrual cycle studies (Cowell et al. 2011; Wadnerkar et al. 2008a); and (b) ten items yielding scores between − 100 (completely left-handed) and + 100 (completely right-handed) (Oldfield 1971) in the menopause study (Maitreyee et al. 2023). The majority of participants were rightwardly lateralised as represented by positive median scores (Table 1) Eighty-nine percent and eighty-six percent of participants from the ‘sex and menstrual cycle’ and menopause studies, respectively, had scores between + 60% and + 100%; this was consistent with the ranges documented in a recent meta-analysis of 200 handedness studies (Papadatou-Pastou et al. 2020).

Data were collected as part of a series of three studies between 2004 and 2016 that investigated the effects of sex, menstrual cyclicity, and menopause on speech, language, and related behaviour. Two studies investigated women longitudinally across menstrual cycle phases and one of these also included men (Cowell et al. 2011; Wadnerkar et al. 2006, 2008a). Data from these two studies (Study 1, N = 45: women, n = 25, men, n = 20; Study 2: women, N = 10, an age-matched subset from the total study) were combined into a single corpus of fifty-five participants for analysis of sex differences and menstrual cycle effects. A separate study investigated women (Study 3, N = 71) from different menopause stages (Maitreyee 2015, 2016). The verbal fluency data from the studies that investigated women longitudinally across the menstrual cycle and men, were not previously published in their entirety. Preliminary reports on performance and strategy for a subset of the data were presented in a conference abstract (Wadnerkar et al. 2008b) and as part of a PhD thesis (Wadnerkar 2008). The verbal fluency data from the study on women and menopause, were not previously published in their entirety. Preliminary reports on performance and strategy were presented in a conference abstract (Maitreyee et al. 2015), as part of a PhD thesis (Maitreyee 2016) and for performance only in a subgroup of postmenopausal women (Maitreyee et al. 2023). Thus, the current analysis reports new results on verbal fluency performance and strategy, for a larger set of combined data.

### Procedures

To control for practice effects, the verbal fluency task was presented in a cross-sectional manner in all three studies. Women from repeated measures menstrual phase studies were tested on the verbal fluency task during only one of the phases. Women from the menopause study and men from the menstrual cycle and sex differences study were tested once.

The calendar method was used to classify the menstrual phases in both menstrual phase studies. The menstrual phase was defined as Days 2–5 and is known to have low levels of both estrogen (E) and progesterone (P), hence was classed as the Low E phase (LEP). An exception to this was that one LEP women was tested on day 6.

The periovulatory phase was defined as Days 8–11 and is known to have high levels of E and low levels of P.

The midluteal phase was defined as Days 18–25 and is known to have high levels of both E and P. Retrospective checks were conducted to corroborate that testing at Days 18–25 was during the midluteal phase. This was done by counting the number of days between testing and onset of the next menstrual cycle, which should be 14 days or less. In this sample (n = 15) women in the midluteal phase at the time of verbal fluency testing had their next menstrual period within 3 to 13 days (mean = 7.67 days, SE = 0.0.74).

Only three women were tested during the periovulatory phase, therefore, women from both higher hormone phases were combined into a single group designated as the High E phase (HEP). This was done to maximise sample sizes for statistical comparison while maintaining a hormone-based distinction between groups.

Session 1 was counterbalanced across the menstrual phase to control for menstrual cycle order impacts on the longitudinal measures. In addition, the time point for conducting the single verbal fluency test was counterbalanced across sessions 1 and 2 for the sex and two-phase menstrual study and across sessions 1, 2, and 3 for the three-phase menstrual cycle study. The resulting breakdown for the current sample is as follows: (a) VF was tested at the LEP for n = 17 women. Of these, VF was tested at the LEP during session 1 for n = 9 women, and VF was tested at the LEP during session 2 for n = 8 women; (b) VF was tested at the HEP for n = 18 women. Of these, VF was tested at the HEP during session 1 for n = 12 women, and VF was tested at the HEP during session 2 for n = 6 women.

The mean menstrual cycle length for all women in the combined menstrual cycle dataset was 29.16 days, SE = 0.43 (minimum = 23 and maximum = 35 days).

Classifications in relation to menopausal stages were designated for women in the menopause study (Table 1). Premenopausal women reported having monthly cycles, but with some subtle changes in flow volume and cycle duration. Perimenopausal women reported having irregular cycles with delays in onset and occurrence. Women in the pre- and peri-menopausal stages were combined into a single group, based on their status of not having met the post-menopausal criteria. Post-menopausal women whose menses had paused for at least one year formed a second group. Definitions of menopause stages used to classify women’s status were based on verbal reports, followed published guidelines (Harlow et al. 2012; WHO 1996) and are summarised in Table 1. All postmenopausal women had attained natural menopause after the age of 40; those with hysterectomy alone or hysterectomy with unilateral/bilateral oophorectomy were excluded (Harlow et al. 2012; Jewelewicz and Schwartz 1986; Laughlin and Thorney Croft 2003).

#### Verbal fluency data collection and scoring

The letter based verbal fluency task requires participants to produce as many words as possible beginning with letters F, A, and S in a time span of one minute, for a total of three one-minute trials per person. Participants were instructed not to produce proper names or morphological variants of the same word (e.g., sleep, sleeping).

Testing was done in a quiet room. Responses were recorded onto a digital recorder (Marantz Portable Professional Solid State Recorder, Model PMD670) using a sampling rate of 44,100 Hz. Audio files were then transferred onto Adobe Audition (version 1.5, Adobe Systems Incorporated) (menstrual cycle and sex difference data) or Praat software (Boersma 2001; Boersma and Weenink 2023) (menopause data) for later transcription. The audio files were stored as PCM.wav files.

Three scores were calculated for each letter trial. These included the total number of correct words generated (excluding errors and repetitions), mean cluster size and the number of switches (Troyer et al. 1997). Clusters were defined as groups of consecutively generated words that began with the same first two letters (e.g., about, abyss), words that differed only by a vowel (e.g., fit, fat, foot), words that rhymed (e.g., sand, stand), or were homophones (e.g., sun, son). Words were classified as homophones if clarified by the participant during word production, by defining or spelling the words aloud. The cluster size was calculated beginning with the second word in each cluster. Thus, a cluster with two words got a score of one; a cluster with three words got a score of two and so on. Switches were calculated as the total number of transitions between clusters, including single words for each of the trials. Errors and repetitions were removed from total word counts but were included in calculations of cluster size and switching (Troyer et al. 1997). Total words, number of switches and mean cluster size were computed within each letter trial for use in analyses conducted as a function of the variable Letter; the individual letter values were averaged across the three letter trials for use in tables and analyses that were not conducted as a function of Letter.

#### Inter-rater reliability

Raters were either authors (MWK, RM, PEC) or doctoral students with linguistics backgrounds (AI, RH—see Acknowledgements) from the University of Sheffield. Inter-rater reliability checks were conducted within each of the three studies that contributed data to the current investigation. Pearson’s correlations were used to assess inter-rater reliability, along with intraclass correlation coefficients (ICCs). Correlation values for all three studies are provided in the supplementary material (Supplementary Table 1).

For the first menstrual cycle and sex differences study, an independent rater (AI) listened to the original audio files and re-scored the data according to the methods applied by the first rater (MWK) for all 45 participants. Within letter, all but one measure achieved reliability above 0.90 (the exception was mean cluster size for the letter S, r = 0.89). Across letters for the whole of the verbal fluency task, all measures achieved reliability above 0.94.

Given the high reproducibility of the method across raters, a sampling method was used to ascertain inter-rater reliability in the other two studies. For the second menstrual cycle study, data for 12 participants were scored by two raters (RM, PC). Within letter, all but one measure achieved reliability above 0.90 (the exception was mean cluster size for the letter S, r = 0.79). Across letters for the whole of the verbal fluency task, all measures achieved reliability above 0.93.

Similarly, for the menopause study, data for 8 participants were scored by two raters (RM, RH). Within letter reliability above 0.93 was achieved for all but one measure (the exception was switches for the letter S, r = 0.87). Across letters, all measures achieved reliability above 0.97.

#### Design and data-analysis

Preliminary analyses included comparisons of FIQ to confirm between-group comparability. The verbal fluency task was administered cross-sectionally, therefore, the FIQ scores were analysed to rule out any differences in intellectual ability estimates between women at the HEP and LEP phases and men, and between women across the two menopause stages. Results of FIQ ANOVAs are reported in Table 1 and revealed no significant differences between groups.

The original studies of younger adults were powered mainly to detect repeated measures effects across the menstrual cycle within women in the domains of speech production and speech perception. For those measures of interest, medium effect sizes were estimated from existing two-phase menstrual cycle data for the acoustic phonetic measure of voice onset time, and for dichotic listening speech perception scores. The decision to pool the data to achieve a larger combined sample for verbal fluency analysis in the current paper was taken to enable larger datasets for a cross-sectional menstrual cycle comparison. Moreover, it was decided to publish the data from the younger participants together with the data from the older pre/peri- and postmenopausal women to further expand the size of the overall data set and its representation across the reproductive lifespan. Thus, the power analysis for the current study was conducted after joining the smaller datasets from the younger participant groups. These power analyses (presented below) were therefore conducted prior to the combined data analysis, not prior to the study design, for the reasons outlined above. Conducting power analyses for the ANOVAs, together with the reporting of partial eta squared scores from the ANOVA effects, provides the statistical information needed to understand the current results in the context of type II (beta = 0.20) and type I (alpha = 0.05) error probabilities. For consistency and internal comparison purposes, this approach was applied to the menopause dataset.

In the current study, the analytic objective of the main analyses was to examine effects of menstrual cyclicity, sex differences, and menopause on verbal fluency as a performance measure and on the underlying cognitive strategies. Separate repeated measures ANOVAs were conducted to examine differences at the Group level as the between-subjects factor, where the Groups were defined by sex, menstrual phases, and menopausal stages, and at the task level with Letter (F, A, S) as the within-subjects factor. Total words, mean cluster size and total switches were the dependent measures. Power estimates for these analyses were computed using G*Power software (Faul et al. 2009, 2007) and are summarised below in relation to medium and large effect sizes (Cohen 1977; Richardson 2011). Further details of analyses conducted for each comparison are presented in the supplementary material.

Estimates of sample size to achieve 80% statistical power for medium effect sizes (*f* = 0.25 η_p_^2^ > 0.0588) in repeated measures ANOVAs (alpha = 0.05, k = 2, repeated measures = 3, correlation among repeated measures = 0.330, and epsilon = 0.996) was N = 72 for between factor effects and N = 38 for within factor effects and interactions of within and between factors. An N = 30 for between factor effects and N = 16 for within factor effects and interactions of within and between factors would be required to achieve 80% power for large effects sizes (*f* = 0.40 η_p_^2^ > 0.1379). Thus, with the sample sizes of the current datasets, the models were sufficiently powered for medium to large effects from the menopause analysis (N = 71) and elements of the sex difference analysis (N = 55). The models were sufficiently powered for large effects from the menstrual cycle analysis (N = 35).

Pearson’s correlations were used to analyse the inter-relationships between the total words produced, cluster size and number of switches. This was done separately for men and groups of women as defined by menstrual cycle phases or menopause stages.

## Results and interim summaries

See Table 2 for verbal fluency means (SEs) for total words, number of switches, and mean cluster size, and Table 3 for correlations among these three measures, as a function of sex, menstrual cycle phase, and menopausal stage.

**Table 2:** Means (SEs) for total words, mean cluster size and total switches as a function of menstrual phase, sex, and menopause stage across the studies

	FAS Total Words	FAS Mean Cluster Size	FAS Total Switches
Menstrual cycle phases in women and men			
HEP Women n = 18	17.28 (0.95)	1.57 (0.12)	4.89 (0.66)
LEP Women n = 17	14.59 (0.94)	1.20 (0.12)	2.96 (0.58)
Men n = 20	16.30 (0.63)	1.26 (0.09)	3.27 (0.48)
Menopause stages			
Pre/Perimenopause Women n = 19	17.75 (0.81)	1.58 (0.09)	3.77 (0.39)
Postmenopause Women n = 52	16.81 (0.64)	1.51 (0.06)	3.43 (0.30)

Values are averaged across the letters F, A, and S

**Table 3:** Correlation analyses [95% Confidence Intervals] with large effect sizes (r > 0.50) indicated in bold (Cohen 1992) (*p < 0.05; **p < 0.01)

	Total words & Switches	Total words & mean cluster size	Switches & mean cluster size
Menstrual cycle phases in women and men			
HEP Women n = 18	0.848** [0.625, 0.938] *p* < 0.001 0.768** [0.458, 0.912]	0.052 [− 0.382, 0.503] *p* = 0.837 − *0.016 [*− *0.491, 0.466]*	− 0.036 [− 0.498, 0.564] *p* = 0.886 *0.162 [*− *0.343, 0.594]*
LEP Women n = 17	0.577* [0.212, 0.825] *p* < 0.015 0.650** [0.232, 0.866]	0.639** [0.094, 0.845] *p* = 0.006 *0.443 [*− *0.064, 0.768]*	0.331 [0.008, 0.793] *p* = 0.195 *0.436 [*− *0.072, 0.764]*
Men n = 20	0.400 [0.012, 0.728] *p* = 0.080 *0.369 [*− *0.102, 0.705]*	0.612** [0.214, 0.840] *p* = 0.004 0.530* [0.100, 0.793]	0.435 [− 0.049, 0.876] *p* = 0.055 0.599** [0.199, 0.828]
Menopause stages			
Pre/Perimenopause Women n = 19	0.437 [0.027, 0.750] *p* = 0.061 *0.358 [*− *0.129, 0.706]*	0.431 [0.074, 0.757] *p* = 0.065 0.468* [0.003, 0.767]	0.101[− 0.362, 0.502] *p* = 0.680 *0.109 [*− *0.376, 0.547]*
Postmenopause Women n = 52	0.408** [0.175, 0.618] *p* = 0.003 0.474** [0.223, 0.666]	0.506** [0.211, 0.756] *p* < 0.001 0.495** [0.249, 0.681]	− 0.057 [− 0.294, 0.199] *p* = 0.686 − *0.052 [*− *0.327, 0.232]*

Confidence intervals were bias corrected, accelerated, and computed using 1000 bootstrap samples. Pearson correlations that survived Bonferroni corrections for multiple comparisons *p* < 0.05/5 = *p* < 0.01) are underlined. *Spearman’s rank correlation coefficients [95% Confidence Intervals] are provided in italics*

### Sex differences and menstrual cycle phase effects.

#### Repeated measures analyses

In relation to the above stated hypotheses, comparisons of participants aged 20–27 years. were conducted: (a) by sex (comparing all women to men); and (b) by menstrual phase (comparing women in the LEP to those in the HEP group).

ANOVAs with Group (Women n = 35, Men n = 20) as the between group factor and Letter (F, A, S) as the within-subjects factor were conducted for total words, mean cluster size and number of switches.

Total words: Letter was significant (F = 31.95, d.f. = 2,106, *p* < 0.001, η_p_^2^ = 0.376) but Group (F = 0.10, d.f. = 1,53, n.s., η_p_^2^ = 0.002) and Letter x Group (F = 0.60, d.f. = 2,106, n.s, η_p_^2^ = 0.011) were not. Post-hoc comparisons for Letter showed that effects were based on more words for S (mean = 18.76, SE = 0.56) compared to F (mean = 16.09, SE = 0.71) compared to A (mean = 13.42, SE = 0.56) (S > F, t (54 d.f.) = 4.96, *p* < 0.001; S > A, t (54 d.f.) = 8.23, *p* < 0.001; F > A, t (54 d.f) = 4.01, *p* < 0.001); the significance of all three post-hoc comparisons survived Bonferroni adjustment (*p* < 0.017).

Mean cluster size: Letter (F = 4.38, df = 2,106, *p* < 0.01, η_p_^2^ = 0.076) was significant, but Group (F = 0.85, d.f. = 1,53, n.s., η_p_^2^ = 0.016) and Letter x Group (F = 2.50, d.f. = 2,106, n.s., η_p_^2^ = 0.045) were not. Post-hoc comparisons for Letter showed that effects were based on larger mean cluster sizes for F (mean = 1.46, SE = 0.10) compared to S (mean = 1.39, SE = 0.09) and A (mean = 1.18, SE = 0.09). Of these three comparisons, only the F > A contrast was significant (F > A, t (54 d.f.) = 2.46, *p* = 0.017), but it did not survive Bonferroni adjustment (*p* < 0.017).

Total switches: Letter (F = 3.62, df = 2,106, *p* < 0.05, η_p_^2^ = 0.064) was significant, but Group (F = 0.93, d.f. = 1,53, n.s., η_p_^2^ = 0.017) and Letter x Group (F = 0.04, d.f. = 2,106, n.s., η_p_^2^ = 0.001) were not. Post-hoc comparisons for Letter showed that effects were based on more switches for S (mean = 4.60, SE = 0.51) compared to F (mean = 3.55, SE = 0.50) compared to A (mean = 2.96, SE = 0.42). Of these three comparisons, only the S > A contrast was significant (S > A, t (54 d.f.) = 2.84, *p* = 0.006), and it did survive Bonferroni adjustment (*p* < 0.017).

ANOVAs with Group (HEP Women n = 18, LEP Women n = 17) as the between group factor and Letter (F, A, S) as the within-subjects factor were conducted for total words, mean cluster size and number of switches.

Total words: Letter (F = 24.28, d.f. = 2,66, *p* < 0.001, η_p_^2^ = 0.424) was significant, but Group (F = 4.02, d.f. = 1,33, n.s., η_p_^2^ = 0.109) and Letter x Group (F = 0.04, d.f. = 2,66, n.s., η_p_^2^ = 0.001) were not significant. Post-hoc comparisons for Letter showed that effects were based on more words for S (mean = 18.80, SE = 0.78) compared to F (mean = 16.11, SE = 0.98) compared to A (mean = 13.00, SE = 0.76) (S > F, t (34 d.f.) = 4.11, *p* < 0.001; S > A, t (34 d.f.) = 6.50, *p* < 0.001; F > A, t (34 d.f) = 3.49, *p* < 0.001); the significance of all three post-hoc comparisons survived Bonferroni adjustment (*p* < 0.017).

Mean cluster size: Letter was not significant (F = 0.18, d.f. = 2,66, n.s., η_p_^2^ = 0.005). Group (F = 4.78, df = 1,33, *p* = 0.036, η_p_^2^ = 0.126) was significant with women at the HEP (M = 1.57, SD = 0.51) having larger mean cluster sizes than women at the LEP phase (M = 1.20, SD = 0.48) (ES *d* = 0.747). Letter x Group (F = 0.24, d.f. = 2,66, n.s., η_p_^2^ = 0.007) was not significant.

Total switches: Letter was not significant (F = 2.78, d.f. = 2,66, n.s., η_p_^2^ = 0.078). Group (F = 4.78, df = 1,33, *p* = 0.036, η_p_^2^ = 0.127) was significant with women at the HEP (M = 4.89, SD = 2.81) producing more switches than women at the LEP phase (M = 2.96, SD = 2.38) (ES *d* = 0.741). Letter × Group (F = 0.24, d.f. = 2,66, n.s., η_p_^2^ = 0.007) was not significant.

#### Correlational analysis

There were no significant Letter x Group effects, therefore the data were pooled by averaging across Letter for correlation analysis. ANOVA results showed significant effects related to menstrual cycle phase, therefore, women’s data were analysed separately for the HEP and LEP groups. Pearson correlations were computed within each group and compared across the three groups to examine relationships between total words, number of switches and mean cluster size and are presented in Table 3.

**Fig. 1:**
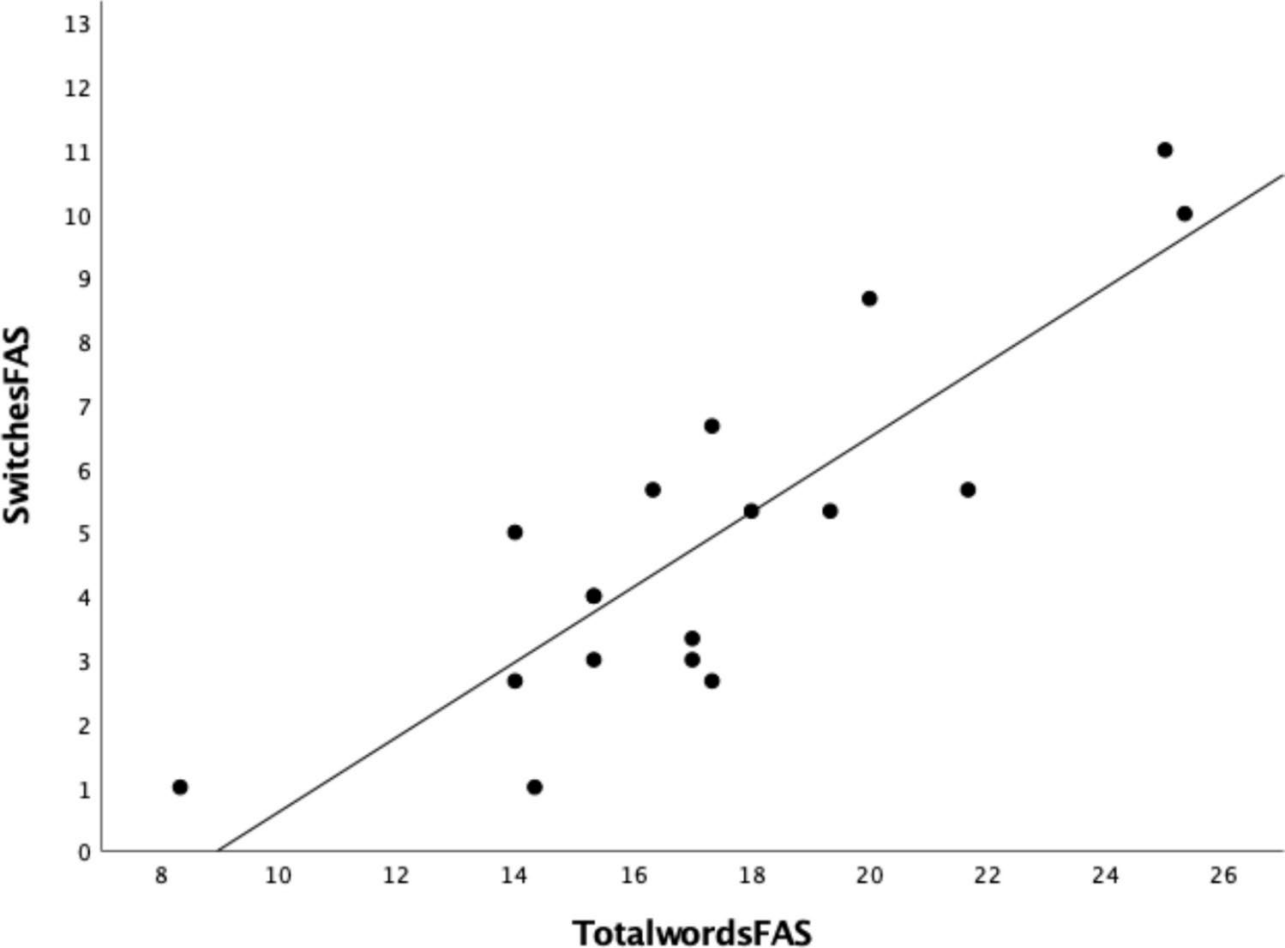
Correlation between number of switches and total words in women at HEP with regression line for r = 0.848 (*p* < 0.001). (SwitchesFAS = number of switches, TotalwordsFAS = total number of words)

In participants aged 20–27 years, both HEP and LEP women had significant correlations between switches and total words that were larger in magnitude compared to men. In contrast, women at the LEP phase and men had significant correlations between cluster size and total words that were larger in magnitude compared to women at the HEP phase. A similar pattern was observed for the correlations between switches and mean cluster size, but without significant correlations for any group. Group differences in correlations were significant for ‘total words to switches’ between HEP women and men (Fisher’s z = 2.35, two-tailed *p* = 0.019). No other between group contrasts were statistically significant for correlations in ‘total words to switches’ or ‘total words to cluster size.’ The correlation between total words and switches for HEP women is depicted in Fig. 1. Fisher-z comparisons are summarised in Table 4.

**Table 4:** Fisher’s z-score comparisons of between group correlations

Total words and Switches	Fisher's z	*p*-Values two-tailed
HEP vs Postmenopause	2.78	0.005
HEP vs Men	2.35	0.019
HEP vs Pre/PeriMeno	2.20	0.028
HEP vs LEP	1.61	0.105

Total words and mean cluster size	Fisher's z	*p*-Values two-tailed
HEP vs LEP	1.91	0.056
HEP vs Men	1.86	0.063
HEP vs Postmenopause	1.71	0.087
HEP vs Pre/PeriMeno	1.14	0.254

Comparisons are summarised by rank for those yielding significant differences, up to and including the nearest threshold for non-significant contrast. The comparison that survived Bonferroni correction for multiple comparisons p < 0.05/4 = *p* = 0.0125) is underlined

**Fig. 2:**
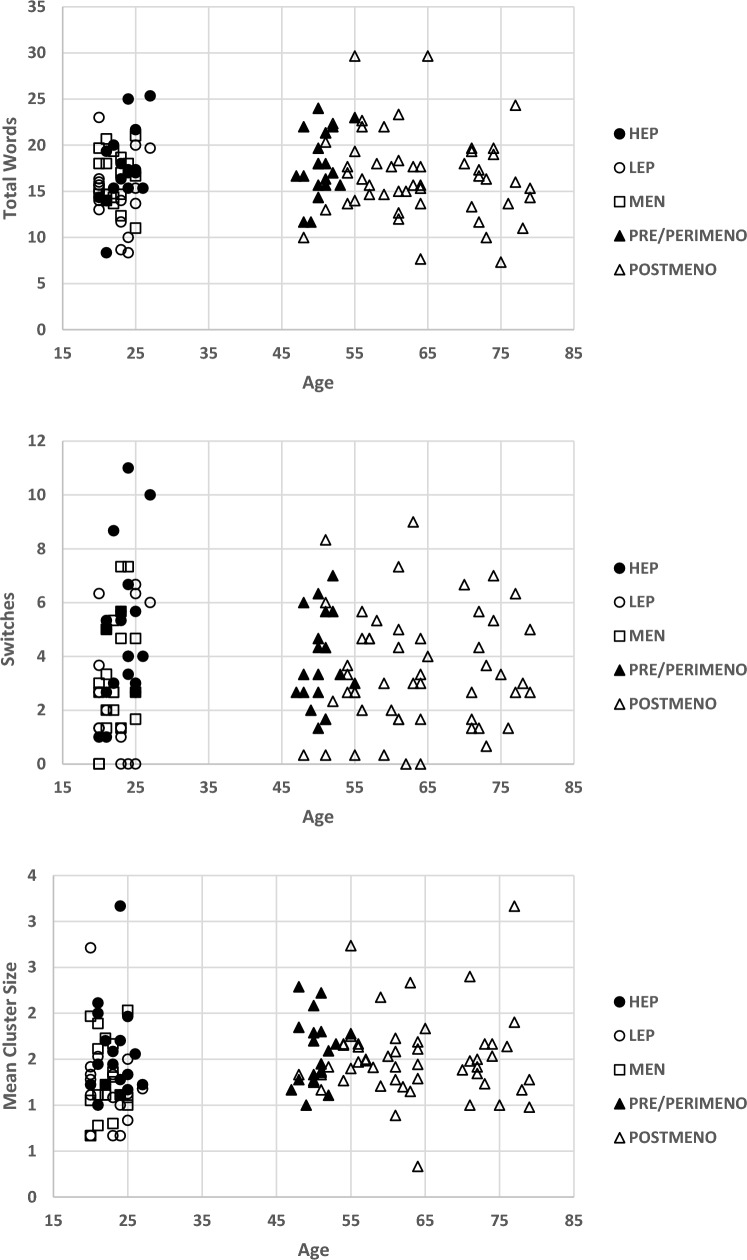
Raw data for all participants as a function of sex, menstrual cycle, and menopause across the three studies. Data for total words (top panel), switches (centre panel) and mean cluster size (bottom panel) are plotted against age in years. (HEP = high estrogen phase; LEP = low estrogen phase; PRE/PERIMENO = pre- and perimenopause stage; POSTMENO = postmenopause stage)

Spearman’s correlations were also conducted to confirm the robustness of the above associations given the degree of naturally occurring variability at the high and low ends of some within-group distributions (See Fig. 2). These aligned closely with the Pearson’s correlations (Table 3).

#### Interim summary

For sex differences, there were no statistically significant main or interaction effects for Sex. In that context, only the mean value difference for the number of switches was in the predicted direction (Women, n = 35, mean = 3.95, SE = 0.46; Men, n = 20, mean = 3.27, SE = 0.48). Although not predicted, non-significant trends for mean cluster size also followed this pattern (Women, n = 35, mean = 1.39, SE = 0.09; Men mean = 1.26, SE = 0.09). A non-significant pattern in the direction opposite to predictions was observed in total words (Women, n = 35, mean = 15.97, SE = 0.70; Men mean = 16.30, SE = 0.63).

Stronger support for the hypotheses was observed with the menstrual phase comparisons between women which showed a significantly higher number of switches and a non-significant trend for more words in the HEP group. In addition, mean cluster size was significantly larger in HEP women. (See Table 2 for means)

With respect to the task, Letter based differences were significant for all sex-based analyses with the pattern of $S\ge F>A$ for number of words, mean cluster size and number of switches. Between women, letter based differences showed the same $S\ge F>A$ pattern which was significant only for total words. Thus, letter based effects were more evident for total words than to cluster size and switches.

Results from the correlations supported the hypothesis that total words would be more highly correlated with number of switches in women compared to men. The prediction that switches would be more strongly associated with the menstrual cycle HEP than the LEP was also supported. Indeed, the HEP women had a significantly higher correlation than the men. Another key distinction between cycle phase x sex groups was observed between total words and cluster size, where HEP women showed lower correlations compared to LEP women and men.

The new finding from this study was that the profiles of correlations between strategy-based measures (clusters *versus* switches) and total words differed depending on sex and cycle phase. Women at the HEP maximised their word count with switching more than clustering behaviours. The contrast between the two correlations (Total words and switches, r = 0.848; Total words and cluster size, r = 0.052) was distinct among the three young adult groups. Women at the LEP used both strategies with a more balanced combination of switching and cluster size. Men maximised their word count by using larger clusters. Table 5 presents these effects by the rank order of correlations between total words and switches; this highlights the relationships between switching and cluster size correlations across the groups in a series of directional comparisons between strategy types.

**Table 5:** Correlation analyses arranged by rank order of relationship magnitude between total words and switches with large effect sizes (r > 0.5) indicated in bold (Cohen 1992)

	Total words & switches	Total words & mean cluster size	Directional comparison of correlations with total words
HEP Women n = 18	0.848** p < 0.001	0.052 *p* = 0.837	switches > cluster size
LEP Women n = 17	0.577* p < 0.015	0.639** *p* = 0.006	switches < cluster size
Pre/Perimenopause Women n = 19	0.437 *p* = 0.061	0.431 *p* = 0.065	switches = cluster size
Postmenopause Women n = 52	0.408** *p* = 0.003	0.506** p < 0.001	switches < cluster size
Men n = 20	0.400 *p* = 0.080	0.612** *p* = 0.004	switches < cluster size

Uncorrected *p*-values are presented (two-tailed significance *p < 0.05; **p < 0.01). Correlations that survived Bonferroni corrections for multiple comparisons p < 0.05/5 = p < .01) are underlined

### Menopause effects

#### Repeated measures analysis

ANOVAs with Group (Pre/Perimenopause n = 19, Postmenopause n = 52) as the between factor and Letter (F, A, S) as the within-subjects factor were conducted for total words, mean cluster size and number of switches. Given the span of ages within and across the groups (see Table 1), Age as a correlate to verbal fluency measures was examined prior to analysis to determine the need for covariance in the model.

Total words: Age did not correlate significantly with letter fluency for individual letters or across all letters for total words within groups (all letters: pre/perimenopause: r = 0.35, n.s.; postmenopause, r = − 0.19, n.s.) or across groups (all letters: r = − 0.18, n.s.), so was not added as a covariate to the ANOVA.

Letter was significant (F = 18.47, d.f. = 2,138, *p* < 0.001, η_p_^2^ = 0.211) but Group (F = 0.65, d.f. = 1,69, n.s., η_p_^2^ = 0.009) and Letter x Group (F = 0.29, d.f. = 2,138, n.s., η_p_^2^ = 0.004) were not. Post-hoc comparisons for Letter showed that effects were based on more words for S (mean = 19.28, SE = 0.59) compared to F (mean = 16.35, SE = 0.60) and A (mean = 15.56 SE = 0.62); two of these post-hoc comparisons were significant (S > F, t (70 d.f.) = 5.55, *p* < 0.001; S > A, t (70 d.f.) = 6.16, *p* < 0.001) and both survived Bonferroni adjustment (*p* < 0.017).

Mean cluster size: Age did not correlate significantly with letter fluency for individual letters or across all letters for mean cluster size within groups (all letters: pre/perimenopause: r = 0.10, n.s.; postmenopause, r = 0.03, n.s.) or across groups (all letters: r = − 0.02, n.s.), so was not added as a covariate to the ANOVA.

Letter was significant (F = 4.51, d.f. = 2,138, *p* < 0.05, η_p_^2^ = 0.061) but Group (F = 0.31, d.f. = 1,69, n.s., η_p_^2^ = 0.004) and Letter × Group (F = 1.35, d.f. = 2,138, n.s., η_p_^2^ = 0.019) were not. Post-hoc comparisons for Letter showed that effects were based on more words for S (mean = 1.66, SE = 0.07) and F (mean = 1.61, SE = 0.08) compared to A (mean = 1.32, SE = 0.07);); two of these post-hoc comparisons were significant (S > A, t (70 d.f.) = 3.71, *p* < 0.001; F > A, t (70 d.f.) = 2.85, *p* < 0.01) and both survived Bonferroni adjustment (*p* < 0.017).

Total switches: Age did not correlate significantly with letter fluency for individual letters or across all letters for switches within groups (all letters: pre/perimenopause: r = 0.12, n.s.; postmenopause, r = 0.06, n.s.) or across groups (all letters: r = 0.003, n.s.), so was not added as a covariate to the ANOVA.

Letter (F = 1.69, d.f. = 2,138, n.s, η_p_^2^ = 0.024), Group (F = 0.38, d.f. = 1,69, n.s., η_p_^2^ = 0.005) and Letter x Group (F = 0.60, d.f. = 2,138, n.s, η_p_^2^ = 0.009) were not significant.

#### Correlational analysis

There were no significant Letter × Menopause effects, therefore the data were pooled across Letter for correlation analysis. Pearson correlations were computed within each group and compared across menopause groups to examine relationships between total words, number of switches and mean cluster size.

Both groups showed moderate correlation coefficients between switches and total words and between cluster size and total words (Table 3) which were significant in the postmenopausal group.

Women in the pre/perimenopausal group had similar non-significant correlation coefficients between switches and clusters with total words. In contrast, women in the postmenopausal group showed a slightly stronger correlation between total words and mean cluster size.

Comparisons between pre/perimenopausal and postmenopausal women for ‘total words to switches’ and ‘total words to cluster size’ were not statistically significant.

Spearman’s correlations were also conducted to confirm the robustness of the above associations given the degree of naturally occurring variability at the high and low ends of some within-group distributions (See Fig. 2). These aligned closely with the Pearson’s correlations (Table 3).

#### Interim summary

There were no statistically significant Group or Group x Letter effects. However, the non-significant trends in the mean values followed the predicted pattern of women in the pre/peri menopause stage generating more words and making more switches compared to women in the postmenopausal stage. A similar pattern of means was observed with cluster size.

With respect to the task, Letter was significant for the total words produced and mean cluster size showing the same $S\ge F>A$ pattern observed for the sex and menstrual cycle phase analyses.

Correlations between strategy-based measures (switches *versus* cluster size) with total words did not differ markedly depending on the menopausal stage. Due to a smaller sample size, correlations in women at the pre/perimenopause were not significant. However, women in both pre/perimenopause and postmenopause stages showed moderate correlation coefficient magnitudes with both switching (0.437, 0.408) and clustering (0.431, 0.506) to total word count. However, there was a tendency for switching to correlate less, and for cluster size to correlate more, with total word count in the postmenopause group. Table 5 presents these effects by the rank order of correlations between total words and switches; this highlights the relationships between switching and cluster size correlations across the pre/peri- and postmenopause groups and positions their correlation patterns between those of LEP women and men.

## Discussion

### Mean differences total words, switches, and mean cluster size

Among the group mean differences by sex, menstrual cycle, and menopause stage, only the contrasts between HEP and LEP women reached or neared statistical significance for the three dependent measures of verbal fluency. Indeed, the widest distinctions in performance and strategy measures occurred in the younger women as a function of their menstrual cycle phase, with women at HEP tending to produce more words as well as significantly more switches (as hypothesised) and larger clusters, than women at LEP. With the exception that younger women combined across cycle phases tended to produce fewer words than men, patterns of means for other comparisons, while not statistically significant, were generally in the predicted direction. Younger women tended to show more switches and larger cluster sizes than men, and pre/perimenopausal women tended to show more words, switches, and larger cluster sizes than postmenopausal women. Looking at the pattern of means from the four groups of women, those with higher within-study ovarian hormones (HEP and pre/perimenopausal groups) tended to produce the most words and used, or tended to use, the most switches. Although not specifically hypothesized, these groups also had or tended to have larger within-study cluster sizes compared to LEP and postmenopausal women.

Findings in this report are consistent with the mixed results reported in the literature on letter/phonemic based fluency, where advantages as a function of female sex, high menstrual cycle hormones, and pre-menopausal status were detected, but varied in size and statistical significance (Hirnstein et al. 2022; Hyde 2016; Sundström Poromaa and Gingnell 2014; Weber et al. 2014). Sex differences with women producing more spoken (Costa et al. 2014; Weiss et al. 2006) and written words than men (Scheuringer and Pletzer 2017) have been demonstrated in some, but not in all studies (Halari et al. 2005; Scheuringer et al. 2017; Sokołowski et al. 2020). The current results are also in accordance with previous research which attributed higher estrogen levels to the enhancement of performance in cognitive areas where females typically outperform males, such as fine motor and verbal abilities (Hampson 1990b, a; Hamson et al. 2016; Maki et al. 2002; Wadnerkar et al. 2006; Whiteside et al. 2004). Menstrual cycle effects with women at higher hormone phases showing better verbal fluency scores than at the lower hormone phase have been shown (Hampson 1990a). In a detailed analysis of narrative fluency, Schultheiss et al. (2021) found a more robust female advantage in age groups 9–17 and 18–50 years compared to children below 9 and adults over 50. Effect sizes were small to medium and strongest in the written modality. Thus, the impact of female reproductive hormones on linguistic fluency appears to be both task and life-stage specific. For example, in contrast to the current study’s findings in spoken letter fluency, menstrual cycle effects were not significant for total words, switches or cluster size in written phonemic fluency (Scheuringer and Pletzer 2017). In relation to reproductive stage, and partly consistent with the current study’s findings of non-significant decreases in letter fluency, lower phonemic fluency scores have been observed for total words in women after menopause in some (Berent-Spillson et al. 2012; Weber et al. 2014) but not all studies (Kilpi et al. 2020) or menopausal stage comparisons (Weber et al. 2014).

In relation to strategy use, Weiss et al. (2006) reported that women used more switches in a spoken phonemic fluency task, but Sokolowski et al. (2020) did not find a sex difference. For written phonemic fluency, results were variable and condition specific, with women using more switches unless instructed to use clusters (Scheuringer and Pletzer 2017) but not consistently so (Scheuringer et al. 2017), and not in a neutral version of the task (Scheuringer et al. 2017). For cluster size, Weiss et al. (2006) reported that men used larger clusters than women for spoken fluency, but this was not replicated in other reports of spoken (Sokołowski et al. 2020) or instructed written verbal fluency (Scheuringer and Pletzer 2017).

In the current study, as noted above, only the menstrual cycle comparisons yielded statistically significant, or near-significant, mean differences for total words, mean cluster size and number of switches. These results corresponded to medium effect sizes based on Cohen’s *d* values greater than 0.50 from post hoc mean comparisons and partial eta-squared values greater than 0.0588 from between-group effects in the ANOVAs. However, the between group comparisons with this sample of 35 women were powered at 80% only for large effects. Despite this limitation, the finding of variation in women’s verbal fluency as a function of menstrual cycle phase explains in part the null findings when the data were analysed by sex. The mean values for total words, switches and cluster size in men were between (in terms of rank order) the higher mean values for HEP women and the lower mean values for LEP women (Table 2). These findings, together with evidence from our previous work in speech production and perception (Wadnerkar et al. 2006, 2008a; Whiteside 2004) indicates that sex differences can be obscured by menstrual phase variation in women. This conceptual framework is useful in part for understanding the nature of hormone related variability underlying small effect sizes in sex differences from studies that do not systematically control for menstrual phase. Our results also emphasize the need to study group differences in the relationships between performance and strategy measures as an additional way to investigate underlying cognitive processing differences in the context of sex and hormone-based factors.

### Correlations between total words, switches and mean cluster size

Previous studies have shown significant correlations between the number of switches and total words produced when looking at spoken phonemic verbal fluency (Troyer et al. 1997) and its written counterpart task (Scheuringer et al. 2017). In relation to sex, effects have been mixed. Weiss et al. (2006) showed the same pattern in male and female groups, with significant correlations between switching and total words. However, Scheuringer et al. (2017) showed that women had a larger magnitude of correlation between switches and total words compared to men on written phonemic fluency, and that this distinction was more prevalent under neutral (i.e., standard) and clustering-based instructions than under switching-based instructions. Neither study showed any correlations for men or women between total words and cluster size (Scheuringer et al. 2017; Weiss et al. 2006). Thus, our findings, in relation to correlations between switches and total words in women compared to men, are consistent with, and expand upon Scheuringer et al.’s (2017) results. In letter based fluency, our correlation of 0.848 between total spoken words and switches in HEP women (n = 18) is consistent with Scheuringer et al.’s (2017) correlation of 0.82 between written words and switches in women (n = 19) who were tested during the luteal phase. Our results, based on a design that specifically compares women at the HEP and LEP menstrual cycle phases, adds an important within-sex comparison to this literature.

The correlations between total words, switching and clustering that were statistically significant, and/or representing large effect sizes, were compared. This was done to elucidate patterns in relation to hormonally related effects on cognitive organisation above and beyond those detected with standalone measures of word count, switches, and cluster size. Patterns of correlations emerged with recurring features across the five participant groups as a function of the intersections between sex and hormonal status.

First, switches correlated most highly with total word count in both groups of younger women, but to a lesser extent in both groups of older women and men. When organised by rank order of correlation between total words and number of switches, the degree to which switches correlated with total words followed a continuum consistent with ovarian hormone levels, from highest to lowest, starting with HEP women, followed by LEP women, then pre/perimenopausal women, and postmenopausal women; men had the lowest ranked correlation (Table 5). Compared to HEP women, correlations between total words and switches were significantly lower in the latter three groups. However, only the comparison of correlations between HEP and postmenopausal women withstood Bonferroni corrections.

Second, mean cluster size correlated most highly with total word count in LEP women, followed in rank order by men, postmenopausal women, pre/perimenopausal women, and HEP women (Table 4). The difference in correlations for total words and mean cluster size approached significance only in the comparison between LEP and HEP women (Table 4).

Combining these two features resulted in three profiles (Table 5), whereby correlations with total words were either:

(i) higher for switches, as in HEP women;
(ii) equivalent for switches and cluster size, as in pre/perimenopausal women;
(iii) or, higher for cluster size, as in LEP women, postmenopausal women, and men.

By framing these profiles in terms of sex by hormone interactions, some preliminary interpretations may be drawn. At the HEP of the menstrual cycle, women in their twenties used a switching strategy more than clustering to achieve their total number of words. In the years prior to and during the perimenopause, women in their late forties and early fifties, showed a blended profile of strategy use in achieving their total words. At the LEP of the menstrual cycle and after menopause, women at both life span stages applied clustering to a greater extent than switching toward their total word performance—a pattern also observed for men in their twenties.

### Combined means and correlations

Integration of the patterns of means and correlations was conducted to yield a cross-group synthesis of the findings. These descriptions are provided for conceptual purposes and are based on a combination of significant and non-significant mean differences and correlations. As such, they are exploratory in nature and are provided for the purpose of generating future research hypotheses. HEP women and pre/perimenopausal women had the highest word counts, alongside a pattern of associations where total words-to-switches were more highly or equally correlated in relation to total word-to-cluster size. In contrast, the three groups with the lowest word counts were those that showed higher total words-to-cluster size correlations compared with total word-to-switches correlations, i.e., LEP women, postmenopausal women, and men. It can also be hypothesised that women have a flexible range of strategies that are differentially deployed across hormone phases and stages throughout a woman’s adult life span. The results observed within the younger women, as a function of menstrual phase, are consistent with a neurofunctional system capable of accessing different strategies during different neurophysiological states. This capacity for change in and of itself represents a capability to make use of a variety word finding strategies to complete the same task.

Effective patterns of cognitive switching at the menstrual HEP may be due in part to the activating effects of estrogen. The reduced number of switches in women at the LEP phase is consistent with findings in girls with Turner’s syndrome who exhibited a reduced number of switches compared to healthy controls (Temple 2002). These results are also in line with previously reported facilitating effects of estrogen on executive function (Dunkin et al. 2005; Erickson et al. 2007; Keenan et al. 2001; Reuben et al. 2021; Sundström Poromaa and Gingnell 2014). Together the evidence suggests that elements of executive functioning may play a greater role in verbal fluency performance in adult women during higher hormone days of the menstrual cycle. The capacity for sex differences and menstrual cycle effects in strategy use has also been studied in spatial tasks, from which one may draw conceptual parallels to the verbal domain. Holden and Hampson (2021) found that women and men relied on different cue types in completing a spatial short term memory task. They also showed a correlation between circulating levels of estrogen and the degree of reliance on the favoured categorial cue type used by women. Analogies may also be drawn from spatial maze behaviour, where women integrated two types of cues compared to men who relied mainly on one cue type (Sandstrom et al. 1998). Thus, the current study’s correlation patterns suggest that in letter based verbal fluency, total word counts in men are more closely linked to clustering, whereas total word counts in women may be associated with clustering and/or switching. During the cycle phases when estrogen is high, the neurocognitive mechanisms for deploying flexible switching strategies in verbal fluency may be mediated by ovarian hormone activation of prefrontal cortex.

Postmenopausal women in the current study showed a pattern of correlations similar to what was observed in LEP women, with greater magnitude in the correlation between total words and cluster size, compared to total words and switches. This finding further solidifies the interpretation above that the presence of high circulating levels of ovarian hormones, as observed for women in the HEP of the menstrual cycle, underpins select use of a switching-based strategy for retrieving more words in the verbal fluency task. Additional support for a connection between estrogen and the capacity to deploy an effective switching strategy for verbal fluency, comes from research in postmenopausal women. Switching in verbal fluency involves the cognitive flexibility to break from existing sets, i.e., clusters, and generate word strings with distinct letter patterns (e.g., breaking from the cluster /*split, splinter, splendid, splash/* to produce the word string /*stick, sandal, stutter/*). Changes at menopause may be due in part to reductions in estrogen’s facilitative effects on prefrontal cortical systems involved in regulating cognitive flexibility (Berent-Spillson et al. 2012; Kantarci et al. 2018; Vega et al. 2016). Thus, as a core dimension of executive function (Diamond 2013), cognitive flexibility measured by switching in verbal fluency, may be mediated by hormone replacement therapy (HRT). Reports have shown that estrogen-based HRT is associated with improved executive function measures (Dunkin et al. 2005; Keenan et al. 2001) and related cognitive processes such as attention (Smith et al. 2001) and working memory (Duff and Hampson 2000) in women after menopause (Navarro-Pardo et al. 2018). Indeed, Erickson et al. (2007) found that up to 10 years of HRT use in postmenopausal women was associated with fewer perseverative errors on the Wisconsin Card Sort Task and a lower degree of volume loss in the prefrontal cortical grey matter.

In pre/perimenopausal women, the correlation between total words and switches was similar to those observed in postmenopausal women and men (Table 3). However, only in the pre/perimenopause group was there near equivalence in the correlations for total words and switches (r = 0.437) compared to total words and cluster size (r = 0.431). The idea that late premenopause and perimenopause are part of a transitional stage to a more stable postmenopausal neurocognitive state is supported by behavioural (Weber et al. 2014) and neuroimaging studies (Mosconi et al. 2021). The current study’s data indicate that perimenopausal changes in letter fluency performance may reflect an underlying reduction in cognitive access to patterns of strategy use available to women at the HEP in earlier adulthood. This, in addition to subtle decreases in overall word finding performance, may give rise to the psychological experience of word finding difficulty during menopausal transition stages – even in instances when actual performance may have stabilised.

We note some methodological limitations in interpreting our findings. One is the degree of statistical power afforded by the sample sizes in each of the three group mean comparisons (i.e., ANOVAs). The menopause comparisons were powered at 80% for both medium and large effects. The sex difference and menstrual cycle comparisons were powered sufficiently for large effects, which were consistently observed only for the within-subjects factor, letter, in analyses of total words. Between group differences were observed in the menstrual cycle comparison but at 12.6% (cluster size) and 12.7% (switches) were just below the threshold required for large effects (Cohen 1977; Richardson 2011). Taking this into account, the data analysis was conducted conservatively, using a collection of methods that comport with the sample size, and which also control for the risk of type 1 errors, including: omnibus analyses of variance as the basis for mean differences; statistical corrections for multiple post-hoc comparisons of means and for multiple correlations; presentation of effect sizes for mean difference effects and correlations; bootstrapped correlation computations presented with confidence intervals; and supplemental non-parametric correlations.

A second limitation is the lack of precise temporal response data to support a more granular analysis of the timing of lexical access and word production in relation to clustering and switching cognitive processes. Addressing these issues in future research would enable confirmatory research on the potential distinctions in hormonally based comparisons—in particular, distinctions between of verbal cognition in women at lower hormone phases/stages and men which are likely to have different neuropsychoendocrinological bases.

### Conclusions and implications

Verbal fluency demands speed and accuracy in generating words (Melinder et al. 2005). It also requires organised and efficient word retrieval, where participants must monitor their responses, providing appropriate words and inhibiting inappropriate ones (Azuma 2004; Henry and Crawford 2004). There is a tendency to produce words in groups or clusters which share a common property. When one cluster is exhausted participants may switch to another (Azuma 2004; Hirshorn and Thompson-Schill 2006; Melinder et al. 2005; Troyer et al. 1997). Results of this study, specifically the mean differences and the patterns of correlations showing large effect sizes, suggest that the ability to produce clusters of words and make switches between clusters in a letter-cued and timed spoken verbal task is impacted by menstrual phases and menopausal stages in women.

This unique study, combining results from a series of datasets, examined verbal fluency performance, strategy use, and performance-strategy associations in healthy adults. Results provide insight into the confluence of factors related to sex, menstrual cycle phase, and menopausal stage that shape cognitive strategy. Moreover, the performance-strategy correlations between total word count, switching and clustering highlighted distinctions that have not been previously revealed through examination of verbal fluency word count alone. From a neuropsychological perspective, the patterns of correlations between total words and switching provide an indirect measure of the close interplay between estrogen levels and verbal thought processes in women across menstrual phases in early adulthood, and menopausal stages in mid and later life. The cross-sectional nature of the studies limits definitive conclusions about cause-effect relationships yet provides insight into the dynamic and complex interplay of sex differences, hormone effects, and reproductive life stages studied here. Further research will be required to more precisely characterise the hormonally mediated neurocognitive mechanisms that influence switching and clustering strategy use in verbal fluency.

On the government, employment, and workplace policy front, there is a growing acknowledgement of the socioeconomic and occupational impact of the menstrual cycle and menopause (GOV_UK 2017; Griffiths et al. 2010; Riach and Jack 2021; Whiteley et al. 2013).There is also increasing clinical interest in understanding issues such as the impact of the menstrual cycle on mental health (Jang and Elfenbein 2019) and of menopause and estrogen exposure on cognitive wellbeing in later life, particularly in the context of aging, acquired and progressive neurological conditions such as dementia (Gurvich et al. 2018; Jett et al. 2022; Paganini-Hill et al. 2020; Rahman et al. 2019; Ryan et al. 2009; Taylor et al. 2019). Indeed, there is an urgency to understand the impact of menopause on women’s health with global rises in people over 60 years of age (Jett et al. 2022; Nebel et al. 2018; UN 2020; WHO 1996). These applied spheres of social and biomedical activity heighten the need for research designed to generate new knowledge on how sex, hormones, and related reproductive factors shape human neurocognitive profiles in people of all ages. In this context, the current study highlights the importance of looking beyond surface measures of behavioural performance to more fully appreciate the variability in cognitive processes employed by women at different phases of the menstrual cycle, stages of menopause, and ages of adult life. Harnessing this variability is key to the design of neuropsychological assessments, and ultimately to the development of policies and practices, that are sensitive and responsive to the distinct cognitive profiles of women and men.

## Supplementary Information

Below is the link to the electronic supplementary material.

Supplementary file1 (DOCX 33 kb)
