# Supplementary material for: Cognitive strategy in verbal fluency: sex differences, menstrual cycle, and menopause effects
Source: Cogn Process. 2025 Apr 5;26(3):641–61. doi: 10.1007/s10339-025-01265-w (PMC12339591; doi:10.1007/s10339-025-01265-w)
Supplement: Supplementary file 1 — Supplementary file1 (DOCX 33 kb) [file 10339_2025_1265_MOESM1_ESM.docx]

**Supplementary Material**

Supplementary Table 1. Inter-rater reliability using Pearson’s correlations (intraclass correlations coefficients in brackets) as a function of data source, letter or whole task, and dependent measure.

| Study data source | Letter / Task | Total Words | Switches | Mean Cluster Size |
| --- | --- | --- | --- | --- |
| First menstrual cycle and sex differences study, data compared for all N = 45 cases | F | 0.990 (.99) | 0.914 (.95) | 0.940 (.96) |
|  | A | 0.987 (.99) | 0.960 (.98) | 0.929 (.96) |
|  | S | 0.974 (.99) | 0.947 (.97) | 0.890 (.94) |
|  | FAS | 0.990 (.99) | 0.985 (.98) | 0.945 (.96) |
| Second menstrual cycle study, data compared for n = 12 subsample of cases | F | 0.987 (.98) | 0.978 (.96) | 0.980 (.97) |
|  | A | 0.996 (.99) | 0.974 (.98) | 0.904 (.88) |
|  | S | 0.989 (.99) | 0.997 (.99) | 0.786 (.76) |
|  | FAS | 0.997 (.99) | 0.975 (.97) | 0.933 (.89) |
| Menopause stage study, data compared for n = 8 subsample of cases | F | 0.990 (.99) | 0.980 (.98) | 0.937 (.94) |
|  | A | 0.943 (.94) | 1.000 (1.00) | 1.000 (1.00) |
|  | S | 0.980 (.98) | 0.872 (.88) | 0.976 (.98) |
|  | FAS | 0.979 (.98) | 0.980 (.98) | 0.979 (.98) |

Supplementary power estimate calculations.

Sex differences: Estimates of sample size to achieve 80% statistical power for medium effect sizes (*f* = 0.25 η_p_^2^>0.0588) in repeated measures ANOVAs (alpha = 0.05, k = 2, repeated measures = 3, median correlation among repeated measures = 0.330, and median epsilon = 0.976) was N=72 for between factor effects and N=38 for within factor effects and interactions of within and between factors. An N=20 for between factor effects and N=12 for within factor effects and interactions of within and between factors would be required to achieve 80% power for large effects sizes (*f* = 0.40 η_p_^2^>0.1379).

Menstrual cycle phase: Estimates of sample size to achieve 80% statistical power for medium effect sizes (*f* = 0.25 η_p_^2^>0.0588) in repeated measures ANOVAs (alpha = 0.05, k = 2, repeated measures = 3, median correlation among repeated measures = 0.332, and median epsilon = 0.996) was N=72 for between factor effects and N=36 for within factor effects and interactions of within and between factors. An N=26 for between factor effects and N=16 for within factor effects and interactions of within and between factors would be required to achieve 80% power for large effects sizes (*f* = 0.40 η_p_^2^>0.1379).

Menopause stage: Estimates of sample size to achieve 80% statistical power for medium effect sizes (*f* = 0.25 η_p_^2^>0.0588) in repeated measures ANOVAs (alpha = 0.05, k = 2, repeated measures = 3, median correlation among repeated measures = 0.226, and median epsilon = 0.998) was N=64 for between factor effects and N=42 for within factor effects and interactions of within and between factors. An N=26 for between factor effects and N=18 for within factor effects and interactions of within and between factors would be required to achieve 80% power for large effects sizes (*f* = 0.40 η_p_^2^>0.1379).
